# The Role of miRNAs in the Regulation of Pancreatic Cancer Stem Cells

**DOI:** 10.1155/2016/8352684

**Published:** 2016-02-24

**Authors:** Sabrina Bimonte, Antonio Barbieri, Maddalena Leongito, Giuseppe Palma, Vitale del Vecchio, Michela Falco, Raffaele Palaia, Vittorio Albino, Mauro Piccirillo, Alfonso Amore, Antonella Petrillo, Vincenza Granata, Francesco Izzo

**Affiliations:** ^1^Division of Abdominal Surgical Oncology, Hepatobiliary Unit, National Cancer Institute “G. Pascale Foundation”, IRCCS, 80131 Naples, Italy; ^2^Animal Facility Unit, National Cancer Institute “G. Pascale Foundation”, IRCCS, 80131 Naples, Italy; ^3^Division of Radiology, National Cancer Institute “G. Pascale Foundation”, IRCCS, 80131 Naples, Italy

## Abstract

Pancreatic ductal adenocarcinoma is currently one of the deadliest cancers with low overall survival rate. This disease leads to an aggressive local invasion and early metastases and is poorly responsive to treatment with chemotherapy or chemoradiotherapy. Several studies have shown that pancreatic cancer stem cells (PCSCs) play different roles in the regulation of drug resistance and recurrence in pancreatic cancer. MicroRNA (miRNA), a class of newly emerging small noncoding RNAs, is involved in the modulation of several biological activities ranging from invasion to metastases development, as well as drug resistance of pancreatic cancer. In this review, we synthesize the latest findings on the role of miRNAs in regulating different biological properties of pancreatic cancer stem cells.

## 1. Introduction 

Pancreatic ductal adenocarcinoma (PDAC) is currently the fourth leading cause of cancer death in the United States and the seventh worldwide, with low overall survival rate [1, 2]. Surgical resection remains the only curative therapeutic treatment for this aggressive disease, although the minority of the patients can undergo resection as consequence of tardive diagnosis [3, 4]. PDAC leads to an aggressive local invasion and early metastases, and it is noted that this disease is poorly responsive to treatment with chemotherapy or radiation therapy [5–7]. To date, gemcitabine is the best chemotherapeutic agent used for pancreatic cancer treatment, although patients showed drug resistance over the time [8–11]. In order to improve PDAC prognosis and to bypass the problem of pancreatic tumor chemoresistance, many alternative treatments have been proposed [12]. Unfortunately, the results are not very encouraging, since the overall survival of patients was not significantly improved. Emerging studies showed that cancer stem cells (CSCs) regulate several mechanisms underlying drug resistance, carcinogenesis, and metastases development in various types of cancer including pancreatic cancer, highlighting the possibility that these cells could represent valid candidates to ameliorate pancreatic cancer prognosis [13, 14].

CSCs were identified for the first time in acute myeloid leukemia [15, 16] and then in the solid tumors, including pancreatic cancer [17–19]. These cells are able to differentiate into several cancer cell types [20] and are involved in the initiation, the propagation, and the therapeutic resistance of various types of human cancer [21–25]. Pancreatic cancer stem cells (PCSCs) show the same properties of normal stem cells and can regulate pancreatic tumorigenesis. It has also been demonstrated that these cells can play several roles in the regulation of chemoresistance in pancreatic cancer [22, 26, 27], although the underlying mechanisms are not completely elucidated. Recent studies dissected the role of microRNAs (miRNAs) and PCSCs on the modulation of pancreatic cancer etiology and progression, shedding light on their importance as potential therapeutic targets for pancreatic cancer [17, 28–31]. In this review, we synthesize the latest findings on the role of miRNAs in regulating key biological properties of pancreatic cancer stem cells.

## 2. Characteristics of Pancreatic Cancer Stem Cells (PCSCs) 

**Figure 1:**
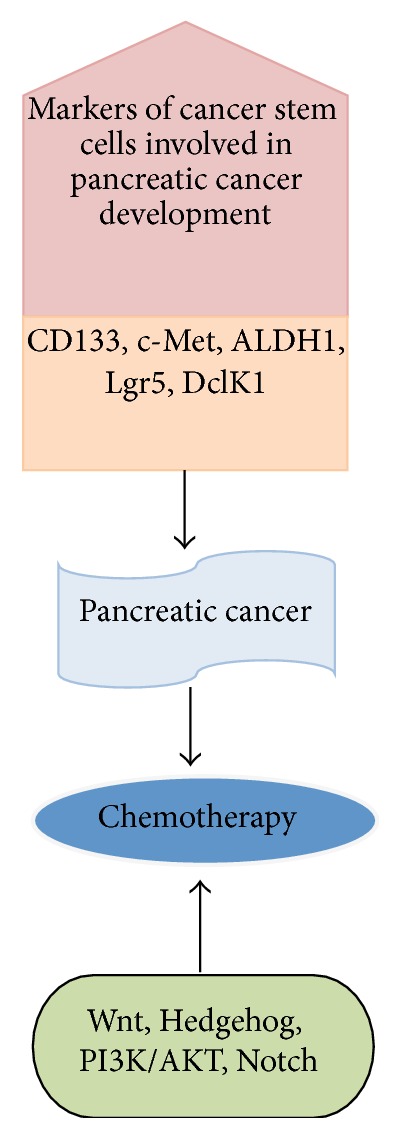
The role of cancer stem cells markers during pancreatic cancer development. The cartoon recapitulates the role of cancer stem cells markers during pancreatic cancer development and the signaling pathways involved.

PCSCs, also named tumor-initiating pancreatic cancer stem cells, were described by Li et al. [22], through the generation of a mouse model of human pancreatic adenocarcinoma. The authors isolated these subsets of cells as CD24^+^CD44^+^ESA^+^ (epithelial specific antigen), which, despite accounting for less than 1% of all pancreatic cancer cells, showed a highly tumorigenic potential respect to noncarcinogenic cancer cells. Later on, many studies based on several xenograft models identified other markers for pancreatic cancer stem cells such as CD133, c-Met, ALDH1 (aldehyde dehydrogenease-1), Lgr5 (leucine-rich repeat-containing G-protein coupled receptor 5), and DclK1 (doublecortin-like kinase 1) (Figure 1) [17, 47–54], although further studies will be necessary to better define the cell surface markers of PCSCs.

## 3. Signaling Pathway Involved in the Regulation of Pancreatic Cancer Stem Cells (PCSCs)

The properties of PCSCs have been investigated by dissecting the underlying signaling pathways and regulatory genes such as Wnt/*β* catenin, Notch, c-myc, Sonic Hedgehog, and Bmi-1 [32, 47, 55, 56] (Table 1). The Hedgehog signaling (Hh) pathway plays a key regulator role in the embryonic development and patterning [33, 57] and it is activated by a complex mechanism of interaction between three Hedgehog (Hh) ligand proteins (Sonic, Indian, and Desert Hh) and cell surface receptors patched (Ptch1 and Ptch2) [13]. It is of note that Hh signaling is necessary for the PCSCs renewal and the maintenance of stemness [58–63]. Notch signaling controls pancreatic self-differentiation by acting on the self-renewal process [64]. Moreover, it has been demonstrated that Notch-1 is involved in the epithelial-mesenchymal transition (EMT) of Aspc-1 pancreatic cancer cell line [65] and in the maintenance of the cancer stem cell population [34]. These studies suggest that Notch signaling is essential for the pancreatic CSC formation. Another signaling involved in the organogenesis of the pancreas is the Wnt-*β*-catenin signaling pathway. It has been demonstrated that Wnt signaling is associated with EMT process in colorectal cancer [66]. Other studies proved that different pathways are involved in the maintenance of pancreatic CSCs such as NF-*κ*B [67] and mTOR pathway [68].

Altogether, these results suggest that different signaling pathways are involved in the self-renewal and the maintenance of pancreatic cancer stem cells.

## 4. The Biogenesis of MicroRNAs (*miRNAs*) 

**Figure 2:**
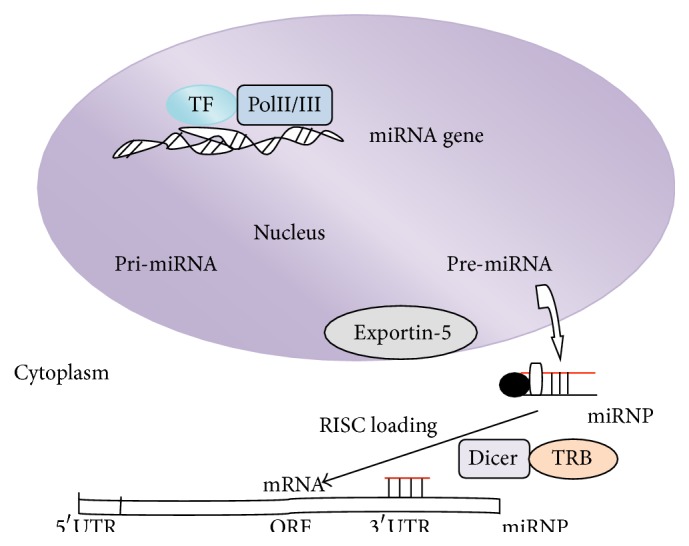
The miRNAs biogenesis.

MicroRNAs (miRNAs) are small noncoding RNAs involved in the regulation of gene expression at posttranscriptional level by binding to the 3′-untranslated regions (3′UTRs) or the open reading frames of target genes. This leads to the repression of mRNA translation or to the degradation of target mRNAs [69]. miRNAs are single-stranded, 18–25 nucleotides long. In animals, they are transcribed as long primary transcripts (pri-miRNA) by RNA polymerase II, which are processed in the nucleus by RNase III Drosha into 70–100-nucleotide-long precursor named hairpin miRNAs (pre-miRNAs) [70]. Then, pre-miRNAs are exported to the cytoplasm by Exportin-5 [71–73] and further cleaved in a complex composed of RNase III enzyme, Dicer, and the transactivating response RNA-binding protein (TRBP) into complex named miRNA:miRNA^*∗*^ [74–77]. The complementary star strand (miRNA^*∗*^) is usually degraded, even if it has been suggested that when it is not degraded, it may have some relevant biological functions [78, 79]. The other strand is chosen as a guide strand that recognizes target mRNAs on the basis of complementarity of sequence. This mature miRNA is loaded into an Argonaute protein within the RNA-induced silencing complex (RISC), which then regulates targets repression by promoting destabilization or inhibiting translation of target mRNA [80–83]. Experimental data showed that miRNAs bind to the open reading frame or to the 5′UTR [84, 85]. The biogenesis of miRNA is showed in Figure 2.

RNA polymerase II transcribes miRNA genes together with specific transcription factors (TF), as long primary transcripts (pri-miRNA). These transcripts are processed in the nucleus by the RNA polymerase III enzyme Drosha, in complex with DGCR8, into pre-miRNAs. These transcripts are exported into the cytoplasm by Exportin-5. Pre-miRNAs are processed by the RNase III enzyme Dicer, in complex with TRBP, into a duplex of a guide strand (miRNA) and passenger star strand (miRNA^*∗*^). The guide stand miRNA is loaded into the RISC and is able to recognize target mRNAs on the basis of sequence complementarity. The RISC regulates target repression.

## 5. The Regulatory Functions of miRNAs on PCSCs Properties

Many studies demonstrated that miRNAs play critical roles in the regulation of CSCs in different types of malignant tumors including pancreatic cancer and have been considered potential targets for cancer therapy, since they are involved in the initiation, the propagation, and the regulation of EMT of cancer stem cells [39, 43, 86–92]. Several miRNAs show different expression profiles in various types of cancer, including pancreatic cancer [93, 94]. Moreover, there is a difference between the miRNA complement of cancer cells and those of nontumor cells. miRNAs can be classified in two different groups based on their role on the progression of human cancer and their expression profile: (1) the oncogenic miRNAs (miR-21, miR-155, miR-17-5p, etc.) that are upregulated in tumor cells [65, 95]; (2) the tumor suppressor miRNAs (miR-34, miR-15a, miR-16-1, let-7, etc.), which are downregulated in pancreatic cancer [43, 96].

Regarding the role of oncogenic miRNAs on PCSCs properties, interesting studies provided evidence that miR-21 modulates the proliferation and the chemoresistance of pancreatic cancer cells [37, 38]. In addition, Giovannetti et al. [97] showed that there is a correlation between miR-21 expression and the clinical outcome of patients with pancreatic cancer through involvement of PI3K/AKT pathway.

On the other hand, other studies showed that upregulated expression of miR-34, which is classified as tumor suppressor miRNA and is regulated by p53, leads to the inhibition of human pancreatic cancer tumor-initiating cells, indicating that miR-34 is involved in the self-renewal process of PCSCs [43, 44].

Hasegawa et al. [46] provided evidence that the overexpression of miRNA-1246 is associated with CCNG2-mediated chemoresistance and stemness in pancreatic cancer.

Studies performed on the expression of various types of miR-200, classified with tumor suppressor miRNAs, demonstrated that these miRNAs can regulate cell maintenance and EMT (by reducing levels of EMT markers) of PCSCs [40].

It has been reported that DCLK1 (a putative marker for pancreatic and intestinal cancer stem cells) regulates EMT in human pancreatic cancer cells through a mechanism dependent on miR-200a [41]. In this paper, the authors demonstrated that DCAMKL-1 expression was upregulated in human pancreatic adenocarcinoma tissue and in a KRAS transgenic mouse model of pancreatic cancer.

Experimental research proved that Zinc finger E-box binding homeobox 1 (ZEB1) is a crucial EMT promoter and inhibits expression of the microRNA-200 (miR-200) family and miR-203, resulting in the maintenance of stemness and EMT activation in colorectal and pancreatic cancer [42].

Pancreatic cancer cell growth can be inhibited also by a complex mechanism of regulation mediated by two tumor suppressor miRNAs, miR-143 and miR-145. Pramanik et al. [45] demonstrated that restoration of miR-143/145 levels, using a systemic nanovector, is able to inhibit pancreatic cancer cell growth in mice. This process seems to be mediated by a negative feedback loop in KRAS/RREB1-miR-143/145. The regulatory functions of miRNAs on the biological properties of PCSs are summarized in Table 2.

Deregulation of miRNAs is also associated with the renewal and the differentiation of stem cells into cancer stem cells, as reported by Garg et al. [98, 99]. Moreover, some important regulators of the stem cell pluripotency (such as Sox9 and Nanog) and miRNAs targets have been described by Ahmed et al. [100].

An experimental approach based on microarray analysis demonstrated a linkage between clusters of miRNAs and clusters of stem cell-associated miRNAs in cancer stem cells [101]. Bao et al. [102] demonstrated that metformin inhibits cell proliferation, migration, and invasion of drug resistant pancreatic cancer cells by attenuating CSC function. This process is mediated by deregulation of miRNAs (let-7a, let-7b, miR-26a, miR-101, miR-200b, and miR-200c) in pancreatic cancer cells.

Altogether, these data suggest that a better comprehension of the regulatory feedback loop between miRNAs and CSC in pancreatic cancer could lead to the development of novel strategies in the treatment of pancreatic cancer patients by CSCs elimination.

## 6. Conclusions

Emerging data summarized in this review showed that miRNAs can function as oncogenes or tumor suppressors, playing important roles in the modulation of several biological activities of PCSCs. Despite these encouraging results, more studies on the function of miRNAs in PCSC biology will be needed in the future in order to improve pancreatic cancer treatments by using miRNAs, as innovative approach.

## Figures and Tables

**Table 1:** Self-renewal pathways in PCSCs.

Signaling pathways	Effects on tumorigenesis and drug resistance	Targets	Reference
Hedgehog	Proliferation	ALDH^+^ CD44^+^CD24^+^ESA^+^	[32–34]
ALK4	Invasion and metastasis	CD133^+^	[35]
Notch	Proliferation	ALDH^+^	[34]
c-Met	Drug resistance	c-Met^+^, CD133^+^	[36]

**Table 2:** The regulatory functions of miRNAs on PCSCs properties.

Upregulation of onco-miRNAs	Downregulation of tumor suppressor miRNAs	Potential targets	Biological PCSCs behavior	Reference
miR-21		*PI3K/AKT*	Proliferation and chemoresistance	[37–39]
	miR-200a/c	*n-cadherin, ZEB 1*	EMT, stemness maintenance	[40–42]
	miR-34	*BCL2*	Self-renewal	[43, 44]
	miR-143/miR-145	*Kras*	Cancer cell growth	[45]
	miR-1246	*CCNG2*	Chemoresistance and stemness	[46]
